# Translation and Validation of the User Version of the Mobile Application Rating Scale Into the Polish Language: Cross-Sectional Methodological Study

**DOI:** 10.2196/65067

**Published:** 2026-01-12

**Authors:** Wojciech Michał Glinkowski, Tomasz Cedro, Joanna Nowicka, Rafał Doniec, Bartłomiej Michalak, Bożena Glinkowska, Stoyan Stoyanov

**Affiliations:** 1 Center of Excellence TeleOrto for Telediagnostics and Treatment of Disorders and Injuries of the Locomotor System Department of Medical Informatics and Telemedicine Medical University of Warsaw Warsaw Poland; 2 Polish Telemedicine and eHealth Society Warszawa Poland; 3 IQCREDO SP. Z O.O. Warsaw Poland; 4 E-statystyka 24 Sonina 360 B by Łańcut Poland; 5 Department of Materials Science and Artificial Intelligence in Dentistry The Medical University of Silesia in Katowice Zabrze, silesia Poland; 6 Department of Biostatistics and Research Methodology Faculty of Medicine, Collegium Medicum Cardinal Stefan Wyszyński University in Warsaw Warsaw, Mazovia Poland; 7 School of Psychology Queensland University of Technology Queensland Australia

**Keywords:** translation, validation, Polish language, mobile health, mHealth, User Version of the Mobile Application Rating Scale, uMARS

## Abstract

**Background:**

Mobile health (mHealth) apps can innovatively diagnose, prevent, and treat many diseases. The increasing use of mHealth apps necessitates an appropriate assessment standard.

**Objective:**

This study aimed to translate the User Version of the Mobile Application Rating Scale (uMARS) into Polish, followed by validation, cultural adaptation, and examination of its reliability and validity.

**Methods:**

The Polish version of uMARS was adapted, translated, and validated based on the free STOP COVID ProteGO Safe app available for Android and iOS platforms. A total of 117 participants rated the app using the translated scale and rerated it 1 week later

**Results:**

The conceptual equivalence of all items and subscales of the original uMARS and its Polish version was confirmed. The translated uMARS scale exhibited high reliability (Cronbach α=0.95). The perceived test-retest reliability and total influence score were acceptable, with intraclass correlation coefficient values of 0.59 and 0.65, respectively

**Conclusions:**

The translated scale matched the reliability of the original scale. It can help users choose the best mHealth apps available in Poland and report their quality. The Polish version of uMARS was cross-culturally validated and was found to be as reliable as the original uMARS. The translated and validated uMARS tool can be used to evaluate mHealth apps in various aspects. App developers can reliably assess app components and determine areas that require further improvement and development to deliver higher-quality apps. The Polish version of the uMARS can become a standard tool for evaluating the quality of mHealth apps in Poland.

## Introduction

Mobile health (mHealth) apps provide innovative approaches [1] to health promotion [2-4], education [5-7], disease diagnosis [8-10], prevention [11], and management [12-15]. In 2017, the number of mHealth apps surpassed 325,000 [16], with an expected annual growth of approximately 41% from 2015 to 2020. The COVID-19 outbreak further accelerated the adoption of digital technologies [17,18], such as telemedicine [10,19-21], eHealth [22,23], and mHealth [9,18,21,24], resulting in an exponential increase in mHealth users [16,25,26].

According to the report by the IQVIA Institute for Human Data Science [27], app stores had approximately 350,000 health-related apps globally in 2020, with more than 90,000 being mHealth apps. The global mHealth app market size is expected to expand at a compound annual growth rate of 11.6% from 2023 to 2030 [27].

In Poland, the number of smartphone users reached 50.6 million in 2021 (132.1 subscriptions per 100 inhabitants), comprising 13.8 and 36.8 million prepaid and postpaid users, respectively [28]. According to a survey by Deloitte Digital [29], in 2022, 75% of the respondents in Poland used mHealth apps, primarily dedicated to fitness, lifestyle, and diet.

Developers, researchers, health care professionals, and web application library managers require appropriate, consistent, and objective tools to assess the quality and safety of mHealth apps. Users frequently choose mHealth apps based on star ratings and short comments in mobile app stores and repositories. Such star ratings may not adequately reflect the quality of the app [30,31]. Generally, user reviews posted on the app download sites provide biased and unreliable descriptions of the apps. These reviews often fail to specify the nature of the app and the circumstances under which it is used and may not cover all helpful features for an accurate evaluation. The traditional star-rating system lacks clarity and is ambiguous in its interpretation. A reliable rating system can help clinicians and patients identify valuable mHealth apps [32-35], streamlining the overwhelming range of available apps to a select few that merit consideration in clinical settings [8,9,14,33,34,36-41]. Many studies have examined the mainstream market status of mHealth apps [16,37,42] by evaluating functional distribution, without refined classifications of mHealth apps and information integrity [1]. In addition, the COVID-19 pandemic complicated the development of many mHealth apps, requiring close attention [18]. The User Version of the Mobile Application Rating Scale (uMARS) targeted this gap by providing an empirical basis for objectively assessing the critical quality criteria and offering users, practitioners, and researchers a rigorous measure of app quality [32]. It helps select and recommend apps [43], owing to growing interest in using objective and sustainable user ratings for mHealth apps, and clinicians and patients can use a measurable, repeatable, clustered evaluation system to ensure that a particular mHealth app can meet their specific needs effectively.

The uMARS provides an empirical basis for the objective evaluation of critical quality criteria. It offers a rigorous metric for evaluating app quality [32] and increasing user confidence [17,32]. With the increasing interest in objective and sustainable user ratings, using tools such as uMARS is well justified. [43].

The considerable international interest garnered by the original uMARS (English) [32] is evident from its translated and validated versions in Italian [44], Japanese [45], Greek [46], Portuguese (Brazil) [47], Spanish [17], and Turkish [48]. Many researchers have highlighted the significance of uMARS as an essential instrument in the evaluation of mHealth apps, with its international use growing continuously [17,35,44-46,49-51]. Although alternative scales [52,53] have been developed in response to the limitations of uMARS [53,54], the uMARS unquestionably fulfills its task and remains the most widespread tool for evaluating mobile apps worldwide [32,33,35,49-51,55-60].

Users’ interest and demand for tools, such as uMARS, are also growing in Poland owing to the increasing number of mHealth apps in the country [61,62]. Consequently, the need for prioritizing individual apps when multiple apps may serve the same purpose has arisen [63].

A translated and validated version of the uMARS was unavailable in Polish despite the increasing number of Polish mHealth apps. Therefore, this study aimed to translate and culturally adapt the uMARS into Polish and test its reliability and validity. The strengths of this study lie in the standardized methods used in all procedures and the rigorous and reliable translation process.

## Methods

### Study Design and Participants

#### Validation Procedure, Participants, and Protocol

We followed the original methodology outlined by Stoyanov et al [32] to validate the final Polish version of uMARS. We assessed the internal consistency and test-retest reliability of the scale.

This cross-sectional methodological study followed STROBE (Strengthening the Reporting of Observational studies in Epidemiology) and COSMIN (Consensus-Based Standards for the Selection of Health Measurement Instruments) guidelines for the validation of patient-reported outcome measures.

The recruitment process focused on the general adult population, and participants who provided written informed consent were considered. The authors recruited volunteers from Polish universities. The inclusion criteria for participants were as follows: individuals aged 18 years or older, individuals who owned and confidently used a smartphone, individuals who were motivated to participate in the study and were fluent in Polish. Participants unable to comprehend or respond to the questionnaires, unable to understand Polish, or unavailable during the study period were excluded.

#### Procedure and Data Collection

##### Questionnaire

The uMARS is a widely recognized tool designed to assess the quality and usability of mobile health apps from the perspective of end users. This study used uMARS to evaluate user perceptions and experiences with a mobile app. The original uMARS comprises 4 objective domains—engagement, functionality, aesthetics, and information—and 1 subjective quality subscale [32]. This structured questionnaire provides valuable insights into an app’s strengths and areas for improvement, ensuring that it meets user expectations and effectively supports the intended health outcomes. By offering a nuanced understanding of user interaction and perception, uMARS contributes significantly to the app’s effectiveness and adoption. Notably, uMARS is used worldwide and does not require special training, making it accessible and practical for diverse research settings.

The uMARS ratings of participants who responded twice per the study protocol were included in the analysis. Singular ratings were excluded from further analysis. The participants evaluated the selected apps. The decision to include only uMARS ratings from participants who responded twice while excluding single ratings was driven by the need to ensure reliability and validity in assessing mobile health apps. The methodology was crucial for ensuring reliability and reducing variability. By requiring participants to rate the app twice, we aimed to increase the consistency and trustworthiness of the collected data. Consistent ratings across the 2 time points helped confirm that user ratings were stable and not influenced by transient factors, such as mood or immediate environment. Excluding single ratings helped reduce the variability resulting from 1-time impressions and ensured that the data reflected a more stable and accurate user experience with the app. The STOP COVID ProteGO Safe app was rated most frequently. Finally, the research team agreed to validate the STOP COVID ProteGO Safe app [64]. The selected app parameters were timeliness, necessity, cost-effectiveness, practicality, and compatibility with Android and iOS platforms.

Considering the assumptions described by the authors of the original method [32], participants received task instructions in the Polish language. They familiarized themselves with the app for at least 10 minutes before providing the ratings using uMARS. This procedure was repeated twice to assess intrarater reproducibility. The sensitive data of the participants were well protected.

##### Adaptation and Translation

The process involved several stages: preparation, double translations from the original version, reconciliation, back translation, review and harmonization, and final proofreading [54,65-69]. Internationally accepted guidelines and checklists [49-51] were used to ensure thorough translation and validation processes [32,52,69]. A bilingual translator professionally translated the English version of uMARS into Polish. The translated output was compared with a validated expert scale. After discussions, minor adjustments were made. Back translation was performed to ensure compatibility and accuracy of meaning between the source and target languages and was reviewed for consistency with the original [49,50]. Finally, the Polish uMARS questionnaire was approved.

##### Validation of the Polish Version of the uMARS

The Polish version of the uMARS questionnaire was validated following the recommendations described by Stoyanov et al [32]. Cronbach α coefficient, which estimates questionnaire homogeneity, was used to measure internal consistency [53,70]. A high internal consistency coefficient was indicated by a value greater than 0.70. Participants completed the translated uMARS questionnaire online and then completed it again after 1 week. The intraclass correlation coefficient (ICC) was calculated to assess the reliability of the translated scale.

##### App Selection

This study was conducted from February 2022 to March 2022, and the STOP COVID ProteGO Safe app was used in this study [64]. The app was freely accessible to Android and iOS users aged 17 years or older.

Similar COVID-19 mHealth apps were available globally [9,18]. The STOP COVID ProteGO Safe app’s Polish version was considered due to its universality and timeliness. The STOP COVID ProteGO Safe app became available in March 2020 [64] and remained active until March 31, 2022. The app received a rating of 4.6 stars for the iOS version (7600 user ratings) and 4.2 stars for the Android version (22,000 ratings from more than 1 million downloads). The official Polish app was developed in collaboration with the Chief Sanitary Inspectorate, which aided the fight against COVID-19. The developer certified the app’s safety, anonymity, data security, and privacy. The app did not collect or share user data with third parties, track the user’s location, or access any files or information on the user’s phone. After the test, the app assessed the user’s most likely COVID-19 risk group based on the guidelines of the World Health Organization and the Chief Sanitary Inspectorate.

### Statistical Analysis

Statistical tests were conducted considering the satisfaction of the assumptions of these tests, including examining whether the distributions of the studied variables conformed to normality. The Shapiro-Wilk *W* test was used to assess the normality of variable distributions. Normal distribution of quantitative variables entailed calculations of mean differences (95% CIs with 1–α=.95 confidence level). External agreement between the 2 measurements was tested for each parameter at *P*<.05. The correlation between each domain and the uMARS total score was assessed using the Pearson correlation coefficient, where a value greater than 0.7 (significance level below 0.05) was considered sufficient to evaluate test-retest reliability. Finally, the ICC between the test and retest was calculated to determine weighted rater agreement values and evaluate the closeness rather than equality of scores.

The reliability of the uMARS questionnaire was assessed via the Student 2-tailed *t* test for dependent variables. The analysis used the final test results and individual categories (results of individual scale parts). Reliability was calculated using Pearson linear correlation and ICC of each variable. Cronbach α (0.95% CI) was calculated to assess internal consistency and reliability. The Cronbach α values were interpreted as excellent (≥0.90), good (0.80-0.89), acceptable (0.70-0.79), doubtful (0.60-0.69), weak (0.50-0.59), and unacceptable (<0.50). Furthermore, the results were supplemented with descriptive statistics (means and SDs).

All analyses were performed using TIBCO Software Inc (version 2017) and Statistica (version 13; Data Analysis Software System) [71]. The results were considered statistically significant at the significance level (α) of .05 if the critical level *P* value was less than .05.

### Ethical Considerations

This study was approved by the Bioethical Commission (AKBE/60/2022). The study was conducted in accordance with the principles of scientific ethics and the Declaration of Helsinki. The Bioethical Commission approved the study protocol on February 21, 2022, and confirmed that the study complies with ethical standards for scientific research. All participants were adults and took part in the study voluntarily. Before participating in the study, participants received information about the purpose and procedures of the study and gave their informed consent in electronic form. Participants were informed that participation in the study was voluntary and that they could withdraw at any time without any consequences. No personally identifiable data were collected. All data were collected and analyzed anonymously. Data storage and processing complied with applicable data protection regulations, and access to the data was restricted to the research team. Participants did not receive any financial or other compensation for their participation in the study.

## Results

### Overview

This study enrolled 117 participants who fulfilled the inclusion criteria (women: n=81, 69.2%; men: n=36, 30.8%; mean age 20, SD 1.66 years). Among them, 90 (76.9%) participants were associated (academically or professionally) in the medical field; 19 (16.2%) were in biomedical engineering, 4 (3.4%) were in nursing, and 4 (3.4%) were in dentistry. Most respondents were undergraduate and postgraduate students enrolled at Cardinal Stefan Wyszyński University in Warsaw (n=39, 33.3%), the Medical University of Warsaw (n=60, 51.3%), and the Silesian University of Technology (n=18, 15.4%). A total of 71 (60.7%) respondents reported daily use of mobile apps, and 59 (50.4%) respondents used Apple, iOS, or other Android devices.

All respondents performed the initial app assessment twice with a 1-week gap using uMARS. The questionnaire was administered twice with a 1-week break to assess the repeatability of the Polish uMARS questionnaire. First, a paired *t* test was conducted to observe the differences between the 2 measurements for each subscore and total score. Test-retest reliability was assessed for each variable using the Pearson 2D correlation coefficient.

The participants rated the STOP COVID ProteGO Safe app twice. Every participant received clear and concise information regarding the study objectives, rules of participation, and procedures.

### Translation Process

The translation process involved several stages: preparation, double translations from the original version, reconciliation, back translation, review and harmonization, and final proofreading [65,72]. Internationally accepted guidelines and checklists [66-68] were used to ensure thorough translation and validation processes [72,73]. Two native Polish-speaking translators independently translated the original English survey into Polish. A consensus meeting agreed upon a standard version as a unified translation. Similarly, 2 independent back translations into English were conducted anonymously using the Polish version of the uMARS. The interdisciplinary group reviewed and completed the questionnaires. No significant difficulties were encountered during the translation process, and a consensus was reached regarding minor discrepancies that reflected the cultural context of the Polish language or semantic issues. The final consensus on the Polish version of the uMARS was achieved after a group of pretesters tested the questionnaire. They confirmed its comprehensibility and transcultural adaptation. A translation history review and examination of the original questionnaire confirmed that all items in the original uMARS were flawlessly translated without major substantive problems. Consequently, the final version was presented to respondents unfamiliar with uMARS, seeking their input on the translated words, understandability, interpretability, and cultural relevance. A ready-to-use version of the Polish uMARS was made available after a final review.

### Test-Retest Reliability

Participants completed the questionnaire twice, providing responses for an initial assessment (test) and a reassessment after a week (retest), considering the values obtained by pairs of the same respondents (*P*>.05; Table 1).

A paired *t* test was performed to compare the results of the test and retest to assess the reliability of the test and retest of the Polish version of uMARS.

The external consistency of the 2 measurements was statistically significant for each measured parameter (*P*<.05). The highest agreement was in category F (perceived impact; *r*=0.73; *P*<.001). The concordance for the total scale score was moderate (*r*=0.40; *P*<.001); however, concordance was not observed for category C12 (visual appeal; *P*=.18; Table 2).

**Table 1:** Sociodemographic and educational characteristics of study participants (N=117).

Domain and subdomain			Paired *t* test				
			Test, mean (SD)		Retest, mean (SD)		*P* value
**A (engagement)**							
	A1 (entertainment)	3.10 (1.16)		3.19 (1.16)		.20	
	A2 (interest)	3.61 (0.94)		3.68 (1.01)		.49	
	A3 (customization)	3.37 (0.87)		3.48 (1.08)		.37	
	A4 (interactivity)	3.45 (0.92)		3.53 (0.93)		.53	
	A5 (target group)	4.09 (0.80)		4.15 (0.88)		.52	
A (engagement total)			17.02 (3.86)		17.29 (4.51)		.58
**B (functionality)**							
	B6 (performance)	4.11 (0.80)		3.99 (0.81)		.11	
	B7 (ease of use)	3.95 (0.85)		3.98 (0.84)		.92	
	B8 (navigation)	3.87 (0.81)		3.90 (0.93)		.91	
	B9 (gestural design)	3.88 (0.78)		3.97 (0.88)		.55	
B (functionality total)			14.90 (3.95)		15.09 (3.87)		.81
**C (aesthetics)**							
	C10 (layout)	4.04 (0.80)		3.92 (0.97)		.17	
	C11 (graphics)	3.96 (0.88)		4.02 (0.84)		.70	
	C12 (visual appeal)	3.83 (0.82)		3.78 (0.79)		.54	
C (aesthetics total)			11.63 (2.67)		11.65 (2.24)		.97
**D (information)**							
	D13 (quality of information)	3.92 (0.80)		4.02 (0.77)		.39	
	D14 (quantity of information)	3.76 (0.76)		3.78 (0.88)		.91	
	D15 (visual information)	3.95 (0.82)		3.92 (0.84)		.48	
	D16 (credibility of source)	4.11 (0.79)		4.21 (0.71)		.45	
D (information total)			14.51 (3.92)		15.02 (3.76)		.12
**Quality**							
	(A+B+C+D)/4	14.47 (2.90)		14.75 (2.98)		.43	
**E (subjective quality)**							
	E17 (recommendation to others)	3.48 (1.14)		3.49 (1.01)		.75	
	E18 (use and relevance)	3.30 (1.30)		3.41 (1.18)		.48	
	E19 (payment)	2.46 (1.23)		2.54 (1.22)		.54	
	E20 (overall rating)	3.62 (0.79)		3.64 (0.87)		˃.99	
E (subjective quality total)			12.56 (3.88)		12.91 (3.59)		.49
**F (perceived impact)**							
	F1 (awareness)	2.72 (1.35)		2.87 (1.23)		.21	
	F2 (knowledge)	2.82 (1.34)		2.81 (1.13)		.85	
	F3 (attitudes)	2.80 (1.36)		2.61 (1.20)		.07	
	F4 (intention to change)	2.88 (1.32)		2.87 (1.27)		.85	
	F5 (help seeking)	2.92 (1.38)		3.02 (1.26)		.40	
	F6 (behavior change)	2.93 (1.32)		2.94 (1.30)		.60	
F (perceived impact total)			16.71 (7.22)		16.51 (5.91)		.39
Total score			85.66 (21.87)		88.05 (18.58)		.29

**Table 2:** Descriptive statistics of total scores and subscale scores of the User Version of the Mobile Application Rating Scale, Polish version (uMARS-PL).

Domain and subdomain			Reliability		
			Pearson correlation		*P* value
**A (engagement)**					
	A1 (entertainment)	0.52		<.001	
	A2 (interest)	0.41		<.001	
	A3 (customization)	0.33		<.001	
	A4 (interactivity)	0.20		.041	
	A5 (target group)	0.27		.004	
A (engagement total)			0.37		<.001
**B (functionality)**					
	B6 (performance)	0.31		.001	
	B7 (ease of use)	0.41		<.001	
	B8 (navigation)	0.49		<.001	
	B9 (gestural design)	0.33		<.001	
B (functionality total)			0.39		<.001
**C (aesthetics)**					
	C10 (layout)	0.44		<.001	
	C11 (graphics)	0.35		<.001	
	C12 (visual appeal)	0.13^a^		.166^a^	
C (aesthetics total)			0.29		.001
**D (information)**					
	D13 (quality of information)	0.35		<.001	
	D14 (quantity of information)	0.45		<.001	
	D15 (visual information)	0.27		.009	
	D16 (credibility of source)	0.24		.02	
D (information total)			0.52		<.001
**Quality**					
	(A+B+C+D)/4	0.39		<.001	
**E (subjective quality)**					
	E17 (recommendation to others)	0.35		<.001	
	E18 (use and relevance)	0.53		<.001	
	E19 (payment)	0.49		<.001	
	E20 (overall rating)	0.30		.001	
E (subjective quality total)			0.37		<.001
**F (perceived impact)**					
	F1 (awareness)	0.61		<.001	
	F2 (knowledge)	0.64		<.001	
	F3 (attitudes)	0.76		<.001	
	F4 (intention to change)	0.67		<.001	
	F5 (help seeking)	0.67		<.001	
	F6 (behavior change)	0.63		<.001	
F (perceived impact total)			0.73		<.001
Total score			0.40		<.001

^a^Statistically significant difference between test and retest scores (*P*<.001).

The reliability analysis of Cronbach α confirmed the very high reliability of the scale at 0.95. No relevant factor lowered or increased the reliability of the entire tool (Table 3).

The ICC values were overall moderate. The highest ICC values were for the category *perceived impact* (ICC=0.59 for 0.29-0.53). The intraclass correlation coefficient for the total uMARS score was moderate (ICC=0.65), indicating moderate temporal stability. This level of agreement is consistent with comparable validation studies of user-rated app evaluation tools, where subjective perceptions of design and engagement may vary naturally between assessments.

**Figure 1:**
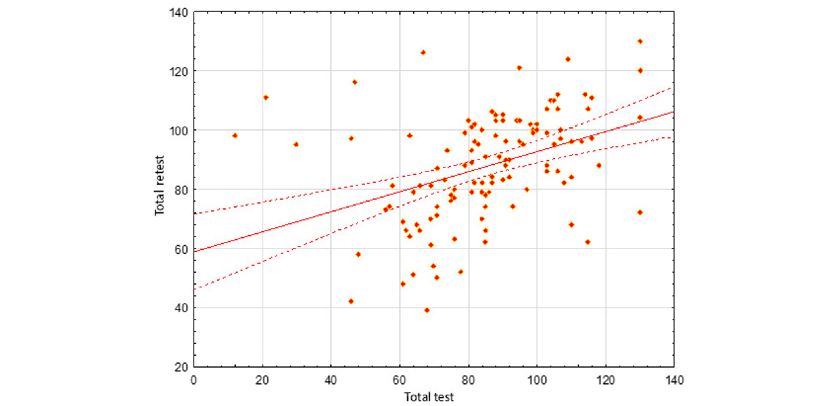
Scatterplot of final test values: total test vs complete retest.

Figure 1 presents a scatterplot depicting final test values (total test vs complete retest). The scatterplot shows the relationship between the overall scale scores on the test and retest. The results were positively correlated, as evidenced by the upward slope of the solid red regression line.

Participants who gave higher ratings on the test tended to give higher ratings on the retest, whereas those who gave lower ratings on the test tended to give lower ratings on the retest. The dots running in an even pattern along the regression line illustrate this observation. The dashed lines indicate CIs of the mean scores.

**Figure 2:**
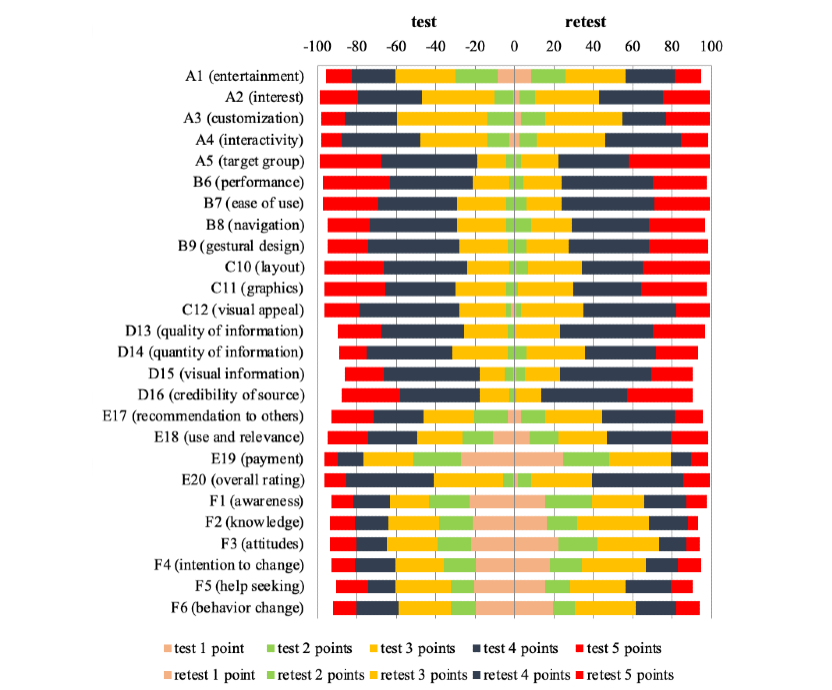
Detailed measurement results of the test and retest.

Figure 2 shows the percentage distribution of individual ratings (1-5 points) provided by the respondents in each category. The left and right sides represent the test and retest results, respectively. Different colors denote each rating. Matching colors indicate specific assessments in both measurements with similar frequencies. The test results mirrored the retest results, thereby forming a mirror image pattern.

The overall ICC values and those for the entire scale were moderate (ICC=0.65). The highest ICC values were for the category *perceived impact* (ICC=0.59 for 0.29-0.53).

**Table 3:** Internal consistency and test-retest reliability of the User Version of the Mobile Application Rating Scale, Polish version (uMARS-PL).

Domain and subdomain			Cronbach α
**A (engagement)**			
	A1 (entertainment)	0.95	
	A2 (interest)	0.95	
	A3 (customization)	0.95	
	A4 (interactivity)	0.95	
	A5 (target group)	0.95	
A (engagement total)			—^a^
**B (functionality)**			
	B6 (performance)	0.95	
	B7 (ease of use)	0.95	
	B8 (navigation)	0.95	
	B9 (gestural design)	0.95	
B (functionality total)			—
**C (aesthetics)**			
	C10 (layout)	0.95	
	C11 (graphics)	0.95	
	C12 (visual appeal)	0.95	
C (aesthetics total)			—
**D (information)**			
	D13 (quality of information)	0.95	
	D14 (quantity of information)	0.95	
	D15 (visual information)	0.95	
	D16 (credibility of source)	0.95	
D (information total)			—
**Quality**			
	(A+B+C+D)/4	—	
**E (subjective quality)**			
	E17 (recommendation to others)	0.95	
	E18 (use and relevance)	0.95	
	E19 (payment)	0.95	
	E20 (overall rating)	0.95	
E (subjective quality total)			—
**F (perceived impact)**			
	F1 (awareness)	0.95	
	F2 (knowledge)	0.95	
	F3 (attitudes)	0.95	
	F4 (intention to change)	0.95	
	F5 (help seeking)	0.95	
	F6 (behavior change)	0.95	
F (perceived impact total)			—
Total score			0.95

^a^Not available.

## Discussion

### Principal Findings

The Polish version of uMARS exhibited excellent internal consistency and good short-term (weekly) test-retest reliability throughout the validation process. The subscales demonstrated either excellent or good reliability in all domains. This version enabled Polish users to obtain reliable measurements of mHealth app quality. The app evaluation system integrated evidence, user experience, and content values into a comprehensive evaluation system. The partial and total results of the assessed domains were reliable and correctly evaluated using the questionnaire.

This study used a single mobile app (STOP COVID ProteGO Safe) as a standardized reference for validation, ensuring linguistic consistency and minimizing contextual bias in participant responses. However, the use of a single app did not permit the assessment of factorial or convergent validity. This limitation aligns with early-phase validation studies of the original uMARS and its adaptations in other languages. Future research should incorporate multiple app categories to confirm construct validity and investigate potential subgroup differences.

The overall internal consistency of the Polish uMARS was high (Cronbach α=0.95). While this supports strong item homogeneity, uniformly high α values across subscales should be interpreted with caution, as they may reflect item redundancy within conceptually overlapping domains. Similar Cronbach α magnitudes have been observed in other uMARS validations and in multi-item satisfaction scales, suggesting that high interitem correlations are typical for this type of measure. Nevertheless, this potential redundancy represents a limitation inherent to user perception instruments. The Polish uMARS questionnaire responds to the growing need for standardization in the evaluation of health-related mobile apps, informed by user feedback. It offers a structured approach to assessing key quality aspects of apps in Polish, benefiting both users and developers.

The app in question is no longer available, as it was designed specifically for use during the COVID-19 pandemic and was withdrawn after it ended. This circumstance makes it impossible to repeat the study using this app, as the app was used only during the COVID-19 pandemic. However, this study aimed to verify the Polish language version of the app. Despite the limited duration of the app’s operation, our goals were successfully achieved, as the aim of this study was to validate the Polish version of the scale.

This validation was conducted on a sample of young university students pursuing health-related education. Although this group was appropriate for the initial phase of linguistic and psychometric validation, it may not fully represent older or less experienced users of digital technology. However, the study group reflects the demographic most engaged in the use of health-related mobile apps. Therefore, focusing on this group was appropriate for initial linguistic and psychometric validation, ensuring that all participants were experienced smartphone users capable of providing reliable app ratings. Future research should evaluate the performance of the Polish version of uMARS among broader and more diverse populations. Validation efforts need to include participants from a wider range of age groups and varying levels of digital literacy to ensure the Polish uMARS’s broader applicability.

### Conclusions

Cross-cultural validation studies of the Polish version of uMARS were conducted, showing its reliability comparable to that of the original version. The Polish version of uMARS can be a valuable tool for assessing the quality of mHealth apps in Poland. From a user perspective, the Polish version of uMARS demonstrates appropriate metric properties for evaluating the quality of mHealth apps, providing comprehensive access to mHealth app ratings from different user perspectives.

Polish researchers can use the translated and verified Polish tool to collect end-user feedback and ratings, helping to identify highly rated apps. In addition, app developers can benefit from reliable app component ratings and gain valuable insights for further improvement and development, increasing the overall quality and impact of mHealth apps. Developers and app stores can present more comprehensive and documented evaluation results along with a star rating to provide users with better insight into the app’s quality.

## Abbreviations

COSMIN: Consensus-Based Standards for the Selection of Health Measurement Instruments
ICC: intraclass correlation coefficient
mHealth: mobile health
STROBE: Strengthening the Reporting of Observational Studies in Epidemiology
uMARS: User Version of the Mobile Application Rating Scale

## Data Availability

The datasets generated and analyzed during this study are not publicly available due to ethical and privacy considerations but are available from the corresponding author on reasonable request.
